# Older care-home residents as collaborators or advisors in research: a systematic review

**DOI:** 10.1093/ageing/afv201

**Published:** 2016-01-19

**Authors:** Tamara Backhouse, Andrea Kenkmann, Kathleen Lane, Bridget Penhale, Fiona Poland, Anne Killett

**Affiliations:** School of Health Sciences, University of East Anglia, Norwich, Norfolk, UK

**Keywords:** patient and public involvement, care home, older resident, PPI, research, systematic review, older people

## Abstract

**Background:** patient and public involvement (PPI) in research can enhance its relevance. Older care-home residents are often not involved in research processes even when studies are care-home focused.

**Objective:** to conduct a systematic review to find out to what extent and how older care-home residents have been involved in research as collaborators or advisors.

**Methods:** a systematic literature search of 12 databases, covering the period from 1990 to September 2014 was conducted. A lateral search was also carried out. Standardised inclusion criteria were used and checked independently by two researchers.

**Results:** nineteen reports and papers were identified relating to 11 different studies. Care-home residents had been involved in the research process in multiple ways. Two key themes were identified: (i) the differences in residents' involvement in small-scale and large-scale studies and (ii) the barriers to and facilitators of involvement.

**Conclusions:** small-scale studies involved residents as collaborators in participatory action research, whereas larger studies involved residents as consultants in advisory roles. There are multiple facilitators of and barriers to involving residents as PPI members. The reporting of PPI varies. While it is difficult to evaluate the impact of involving care-home residents on the research outcomes, impact has been demonstrated from more inclusive research processes with care-home residents. The review shows that older care-home residents can be successfully involved in the research process.

## Introduction

Patient and public involvement (PPI) has developed into an integral part of research practice over the last 25 years. Many funding bodies and ethics committees now require PPI to be part of research protocol development (for example, see Ref. [1]). Research and guidance in this area have considered: the involvement of older adults in research processes [2, 3, **4,** 5, 6, 7]; the participation of marginalised groups in evaluation [**8**]; and enabling research in care homes and working in partnership with them [**9, 10, 11**]. Despite the increasing emphasis on PPI, marginalised groups, such as care-home residents, can be overlooked when including people in the research process.

Care homes in the UK provide 24-h residential care (personal care) or nursing care (personal care and qualified nursing care). Settings vary in size, ownership and specialisms [12]. Groups with a stake in care-home provision include commissioners, owners, managers, staff, residents and relatives. Care-home residents typically have high levels of physical dependency [**13**], three-quarters have cognitive impairment [**14**] and many are nearing the end of their lives, so stay in care homes for relatively short times [**15**]. Consequently, care homes are unique research settings and care-home residents need specific consideration in relation to how they may be involved in research. Recently, guidance has been developed for conducting research in care homes [**16**] and a resource produced to inform researchers planning to involve care-home residents, relatives and friends as PPI members in their research [**17**]. Learning from previous research involving older care-home residents as PPI members is vital to improve the effective inclusion of this marginalised group in future studies so they can have a voice and active role in research.

This systematic review is part of a wider study, Residents Research-Active in Care Homes (RReACH), which aimed to involve care-home residents, older people living in the community and care-home staff as PPI members in collaborator or advisor roles throughout the research (systematic review and interviews with care-home residents and staff). This review therefore aimed to determine how older care-home residents have been involved as PPI members in care-home research.

## Methods

This paper follows the Preferred Reporting Items for Systematic Reviews and Meta-Analyses (PRISMA) guidelines [18]. The researchers also aimed to build a collaborative research team which included older care-home residents and people with an interest in care homes to assist with the review.

### Eligibility criteria

Studies were included if they reported a PPI element involving older care-home residents. To maximise learning, any research topic, methodology, study design or type of PPI was included. Studies were excluded if there was no PPI element, the PPI element did not include residents, the age of the residents was not 65+ and the residential setting did not aim to accommodate older people.

### Information sources

A search of 12 electronic databases (ASSIA, Proquest, AMED, EMBASE, MEDLINE, CINAHL Plus, CINAHL Complete, PsychINFO, PsychArticles, Academic Search Elite, Science Direct and SCOPUS) was conducted in September 2014.

A supplementary lateral search was conducted. For example, searching reference lists of papers and key websites such as INVOLVE and the TRIP database. Searches for related papers and reports of included studies were conducted to try to determine more about the residents' involvement in those studies. When necessary, we contacted authors to attempt to locate further publications.

### Search

The search syntax used was as follows: (Advisory OR client OR engagement OR participatory OR ‘participatory research’ OR ‘patient and public involvement’ OR PPI OR stakeholder OR ‘user involvement’) AND (‘assisted living’ OR ‘care home’ OR ‘elder care’ OR ‘home for the Aged’ OR ‘long-term care’ OR ‘nursing home’ OR ‘Old people's home’ OR ‘residential home’). Limits were set for post-1990, ‘human’ and ‘English’.

### Study selection

**Figure 1.**
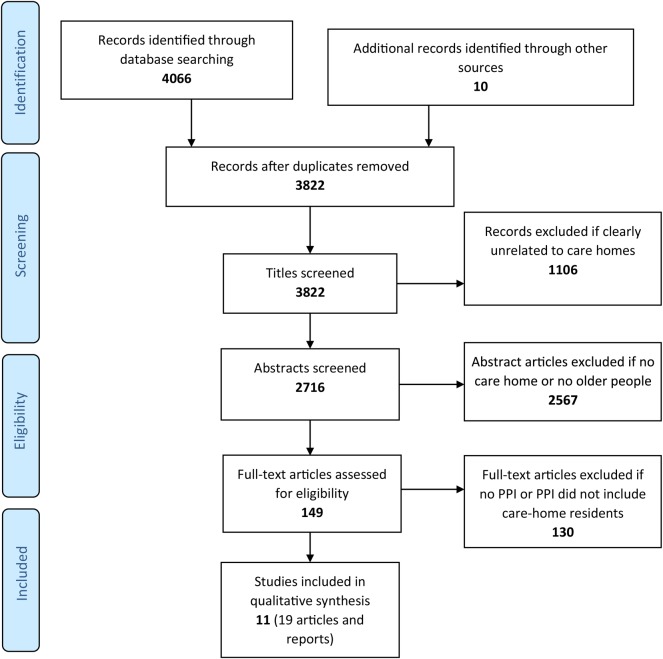
Flow diagram of literature review.

Study selection was undertaken in three stages: first titles were screened, second abstracts were screened and finally full texts were obtained for eligible papers or where eligibility was unclear (Figure 1). Two authors independently carried out the selection process. They made judgements about how studies met the inclusion criteria, disagreements were resolved by discussion and then consensus, a third person was consulted where necessary.

### Data collection process

A data extraction form was developed (Supplementary data, Appendix A, available in *Age and Ageing* online) which enabled the extraction of information relating to PPI elements. The form was piloted by collecting data from two included studies to see whether the extraction categories were plausible and provided useful results. The extraction form was refined and questions relating to: the length of study, the methodology, the direct voice of residents and the decision or advice PPI were involved with added at a project team meeting (which included PPI) before two authors undertook data collection in consultation with each other. Three papers located through supplementary searches [**19, 20, 21**] were included in the review but did not have data extracted as they presented very limited information about PPI.

### Summary measures

The Cochrane Qualitative Research Methods Group chapter on critical appraisal of qualitative research [**22**] was used to inform the development of critical appraisal questions, in the data extraction form. These included the transparency of reporting the PPI process and whether the aims of PPI for individual studies were met. Those scoring low on these quality criteria still offered valuable information in other areas. To maximise understanding of resident involvement, we did not exclude any papers and successful engagement was attributed to studies achieving PPI with older care-home residents.

### Synthesis of results

To synthesise the data, extraction categories were grouped into 13 themes (Supplementary data, Appendix B, available in *Age and Ageing* online) and two authors thematically analysed the data within and across them. Our PPI team members discussed the findings by reviewing the resulting two key themes and providing their thoughts on their plausibility.

### Risk of bias across studies

Because the reporting of PPI in research outputs is non-standardised and selective, the critical appraisal questions in our data extraction form were developed to assess this aspect.

## Results

Database searches identified 4,076 reports and papers, with 19 (relating to 11 different studies) fulfilling the inclusion criteria. All identified studies were included in the review to maximise the knowledge we could obtain.

All 11 studies had a predominantly qualitative research design; however, four also included a quantitative element [**23, 24, 25, 26**]. Table 1 presents information about the 11 studies included in the review. The studies varied in geographical setting, topic and the type of care establishments that they involved. Residents were recruited through the use of written leaflets, existing networks or support meetings, information meetings or staff facilitation.

**Table 1.** Studies involving older care-home residents in the research process

Study	Study topic	Region	Type of PPI	Size of study (*n* = care homes involved)	Publication	Care establishment	Resident demographics
Shura, Siders and Dannefer [**32**]	Culture change in long-term care	USA	Collaboration	Small (*n* = 4)	Paper	Long-term care, assisted living, continuing care retirement community	37 female, 12 male residents with varied levels of physical and cognitive challenges
Cheek *et al.* [**28**]	Use of medicines	Australia	Consultation	Large (*n* = 8 or more)	Paper	Residential care	Not specified
Mitchell and Koch. [**34**]	Giving nursing home residents a voice for quality improvement	Australia	Collaboration	Small (*n* = 1)	Paper	Nursing care	Residents without dementia
Chenoweth and Kilstoff [**23**]	Organisational and structural reform	Australia	Collaboration	Small (*n* = 3)	Paper	Aged-care facilities including nursing and residential and dementia-specific services	Not specified
Aveyard and Davies^a^ [**24**] Davies, Powell and Aveyard [**39**] Froggatt *et al.* [**11**]	Implementation and evaluation of an action group	UK	Collaboration in action group	Small (*n* = 1)	Paper Paper Paper	Nursing home (advanced dementia)	Not specified
Baur and Abma [**35**]	Participation and empowerment through improving the food system in the home	The Netherlands	Collaboration, co-owners of the process	Small (*n* = 1)	Paper	Public residential care home with 129 apartments (56 sheltered accommodation, 73 residential care)	7 female residents aged over 80 with physical disabilities
Hewitt *et al.*^a^ [**33**] Hewitt, Draper and Ismail [**25**]	Food provision in a residential home: intervention and process evaluation	Guyana (researcher a PhD student at a European university)	Residents as participants in focus groups, informal conversations and voting for possible interventions	Small (*n* = 1)	Paper Paper	A residential home for senior	14 residents aged between 73 and 98
Killett *et al*.^a^ [**29**] Burns *et al.* [**40**] Killett *et al.* [**41**] Hyde *et al.* [**19**] Hyde *et al.* [**20**]	Organisational dynamics associated with abuse, neglect and/or loss of dignity of older people in care homes	UK	Consultation and participants (key informants), collaborators	Large (*n* = 8 or more)	Report Paper Paper Paper Paper	Care homes	5 residents, all aged 85 and over
Tadd *et al.* [**26**]	Promoting excellence in care homes by developing a staff training package	UK	Consultation	Large (*n* = 8 or more)	Report	Care homes	Not specified
Killett *et al.*^a^ [**30**] Killett *et al.* [**21**]	Care home cultures of excellence	England, Scotland and Wales	Consultation	Large (*n* = 8 or more)	Report Paper	Care homes (mix of nursing, residential and specialist dementia care)	Not specified
Bowers *et al.* [**27**]	The role and contribution of long-term care within the whole spectrum of future services	England and Scotland	Collaboration (workshop involvement)	Large (*n* = 8 or more)	Report	Long-term care, care home, adult placement locations	Not specified

^a^Key publication from study.

### Two distinct types of involvement

#### Large studies

These five studies involved eight or more care establishments, were multi-method studies and all, but one, were UK based. The topics of the studies varied. One researched the role and contribution of long-term care within the whole spectrum of future services [**27**]. An Australian study explored factors influencing the quality use of medication [**28**]. The other three were part of the research programme, Prevention of Abuse and Neglect in the Institutional Care of Older Adults (PANICOA). The large research projects were set up and led by the researchers, but contained within them different advisory groups or defined elements of collaboration. The researchers aimed to consult with multiple stakeholder groups at various points during the projects, which typically resulted in complex studies.

The involvement of residents was often limited compared with other stakeholders such as care-home staff, relatives or health professionals. In two studies [**26, 29**], care-home residents had partial involvement. For example, residents did not play a part in stakeholder events but could be members of specific advisory groups and panels. Additionally, two studies included peer researchers [**27, 29**], but these were older people from the community, not care-home residents.

When residents were involved in the studies, they were consulted in groups on their own [**29**], with relatives and carers [**26, 30**], or in mixed stakeholder groups [**27, 28**]. Various methods of involvement were employed including workshops [**26, 27**], nominal groups (defined by Delbecq [31]), focus groups [**28**], interviews [**27**] and panel groups [**29, 30**].

#### Small studies

Four of the six small studies involved only one care home, one study involved three care homes [**23**] and one involved four [**32**]. The small studies focused on the immediate care-home environment and emphasised partnerships. The four involving only one care home followed a participatory action design focusing on actual or potential interventions and/or their evaluation. Researchers aimed to collaborate with residents (and others) as partners, involving them in decision-making to examine aspects of the care/service provided. The studies reflecting three or four homes focused on culture change in care homes and involving care-home residents (and others) as co-researchers. The comparative design of these two studies had no input from care-home residents or other parties. Therefore, they shared the same participatory action design as the one-site studies. In such designs, the researcher was viewed primarily as a facilitator [**32**].

All the small studies involved residents and care staff. In addition, most also included as participants, family members and management, while some aimed to involve all people in the care-home community, so including administrative and maintenance staff [**33**] and representatives of the NHS Trust and Housing Association [**24**]. Involvement by the different groups varied at different times. Most studies had a collaborative process; however, the Hewitt study incorporated a more structured approach where residents could vote on 23 potential interventions and prioritise them [**25**].

One small study that took place in a home specialising in dementia care had minimal resident involvement [**24**]. However, this and other small studies made attempts to enhance resident involvement; for example, some used informal conversations to enable residents to be involved [**24, 25**] and others used interviews or focus groups [**23**] as a tool to bring residents' voices to the negotiating table [**34**] or to ensure their participation was maximised [**25**].

### Barriers to and facilitators of resident involvement

Many studies encountered barriers and facilitators of varying types and extent when trying to include residents as PPI members in research, discussed most by the studies focusing on participation, voice or method. Table 2 shows the barriers and facilitators thematically grouped under categories: social factors, skills, resources, care-home organisational factors and the organisation of the research.

**Table 2.** Factors that could be barriers to or facilitators of residents' involvement in research processes

	Barrier and facilitator categories				
	Social factors	Skills	Resources	Care-home organisational factors	Organisation of the research
Barriers	Resident low confidence Apprehension to engage into something different Power relations (mentioned in relation to staff and relatives) Researcher and research seen as threatening (to staff) Frustration about complexity and slow progress Lack of trust in confidentiality Low or changing mood of some residents Role conflict of researching in own home	Sensory and communication difficulties Changing health of residents Cognitive impairment resulting in limited skills to participate and negotiate Meetings might be monopolised by one member Lengthy and complex reports can frustrate residents Residents' low energy	Lack of funding for more continuous input Limited time of the researcher (e.g. not available at the weekend, no time for providing feedback) Lack of space to hold meetings	Unsupportive organisational culture Individuals and groups feel isolated from each other Perception that residents’ involvement might slow down decision-making process Dominant person might influence residents	Limited researcher flexibility Ethical protocols excluded and limited participation Researchers reluctance to relinquish control Timing of meetings, e.g. evening Venue of meeting, e.g. not at care home or lack of privacy
Facilitators	The development of trust and good relationships Residents’ experiences valued Residents are supported to contribute People are open to change Good commitment from the PPI people Transparency of processes Residents have some control, e.g. some ownership over decisions Assurance that the study will result in progress Assured confidentiality Ensure members can stop at any time without reason	Researchers providing constant encouragement and support to residents Researchers embracing deviant perspectives Researchers using successful examples to illustrate involvement Researchers willing to share control Researchers contactable at all times Making negotiated ground rules Being able to communicate with diverse groups of people Researchers using creative methods to engage residents Researchers being flexible	Funding for honorarium for participants Time to do the groundwork required, e.g. proving information Time to arrange meetings and support residents Suitable venues and space to hold meetings Providing sustenance Financial resources to implement changes identified by the research	Supportive organisational culture Care-home management on board Care-home management willing to change Care-home staff value residents being involved in study	Emergent study design Use topics that really matter to the residents Flexibility in residents’ involvement, e.g. use informal conversations if needed Allow personal *ad hoc* contact with research team Summarise meeting notes into accessible formats, e.g. posters Send materials out before meetings Recruit researchers who can support older people Recognise multiple stakeholder groups and support marginalised groups

#### Social factors

The development of good relationships with residents aided involvement. Trust and transparency were important [**11, 23**]; residents' trust in researcher confidentiality could impact on their willingness to be involved [**34, 23, 25**]. Valuing residents' involvement [**32**] and creating a safe space for them to voice ideas could enhance their involvement [**35**]. Residents' confidence levels [**34**] and whether they had a low or agitated mood [**29**] also affected their participation, while some residents and staff could be reluctant to engage with new ideas [**23**].

#### Skills

Resident and researcher skills could impact on resident involvement. Cognitive impairment sometimes presented a barrier, since it meant some residents had poor knowledge and negotiating skills [**23**] or were prevented from taking an active role in meetings [**24**]. Sensory impairments such as hearing difficulties and poor vision could hinder participation [**25**]. To accommodate the changing health of some residents, Killett *et al.* [**29**] held their final meetings with residents in care homes rather than at external venues.

Researchers' communication and interpersonal skills were essential. Investigators had to take on the role of a facilitator or mediator in meetings or negotiations [**34**], foster good relationships with a variety of individuals [**25, 35**] and offer continued encouragement and support to residents [**32**].

#### Resources

Time and money were cited as barriers. In one study, a lack of funds meant that not all residents interested in contributing to the research could be involved [**32**]. Action research required financial resources to allow the implementation of the changes identified [**35**]. Spending time gaining residents' confidence and getting to know them were essential to increasing participation but, due to financial constraints, were difficult to sustain [**24, 34**]. One study did not provide feedback to PPI members at its conclusion due to time limitations [**27**]. Two studies offered care-home residents remuneration for their involvement [**28, 29**].

#### Care-home organisational factors

A supportive organisational culture where management and staff valued the residents' participation in research and were open to change which might enhance involvement and helped the implementation of action research [**23**]. In contrast, fragmented leadership and the attitudes and abilities of key powerful individuals could work against residents' involvement [**25**].

#### Organisation of the research

An emergent design was seen to be important to empower residents, since it allowed them to set the agenda and have some ownership over the study [**35**]. Flexibility, which could similarly encourage residents' involvement, was shown in using informal conversations or one-to-one discussions [**25, 29**]; identifying a main contact or source of support for residents [**27, 29**]; and allowing ad hoc contact with researchers [**24**]. However, allowing ad hoc contact with researchers could be a challenge for research staff to respond to.

## Discussion

This review identified 11 studies that involved older care-home residents as advisors or collaborators. Two distinct types of research involvement with older care-home residents emerged: (i) residents as collaborators in small-scale participatory action research and (ii) residents in advisory roles at certain points on large-scale studies. Multiple barriers to and facilitators of involving residents as PPI members were identified which should be more widely recognised and anticipated in planning residents' PPI. These can be grouped under social factors, skills, resources, care-home organisational factors and the organisation of the research. Flexibility in research designs, processes and ways of practising can enable older care-home residents to have meaningful involvement in research. The review has found that the involvement of older care-home residents in the research process is, indeed, possible. However, until now, few studies have involved this marginalised group and, when included, their contribution was often limited in comparison to other stakeholder groups.

This review adds to the literature by providing knowledge about the involvement of older care-home residents in research processes, based on research experiences. The findings add to the general guidance for conducting research in care homes specific knowledge and learning points about having care-home residents as PPI member. For example, although Luff *et al.* [**16**] discussed resources and flexibility in relation to conducting research in care homes, this review outlines specific ways in which these aspects can facilitate older care-home residents' involvement as PPI members (such as having sufficient resources to develop relationships with residents and allowing ad hoc contact with researchers). This review also augments the knowledge available for researchers who plan to involve care-home residents, relatives and friends as PPI members [**17**], by providing examples and detailed information about how care-home residents have been involved in past research and the barriers to, and facilitators of, their involvement. The high proportion of residents with cognitive impairment and frailty in UK care homes mean that many residents encounter individual-level barriers to involvement. Residents with cognitive difficulties were often screened out from studies or only informally involved. If involved, cognitive difficulties could greatly restrict residents' involvement. Future research should explore the best ways to involve residents with cognitive difficulties in studies, so that their voices can be heard.

Structures surrounding research funding and ethical protocols could inhibit taking some lessons learned from this review forward. For example, some funding bodies are not open to emergent designs, and ethical approval may have to be sought multiple times as a flexible study design takes shape. Additionally, some methodologies are not suited to an emergent design. Therefore, unless residents are involved in the development of a funding bid, it may be difficult to enable them to have much control over the research design.

### Strengths and limitations of the review

Given the nature of the review focus on what is usually a secondary aspect of published research (PPI), it was often difficult to determine whether any involvement had taken place in studies. Consequently, the main limitation of this review is that PPI is not always reported in study outputs; therefore, the findings may not reflect the total range and scope of older care-home residents' involvement in the research process. There was also a blurring between PPI members and participants in some studies, and the processes of resident involvement were not always stated or clearly explained. This presented difficulties in determining the exact nature of resident involvement. Additionally, the impact of resident PPI on the studies was not formally evaluated [36, 37, 38] or clearly reported, making it difficult for the review to assess this element. Due to inconsistent and partial PPI reporting, we found some of the critical appraisal questions difficult to apply. The papers often did not state the aims of PPI and the impact of PPI on the study. Therefore, a full understanding of the value of PPI with this population could not be determined. Consequently, for this review, the achievement of involving older care-home residents as PPI members constitutes success.

Throughout the systematic review, we intended to involve our own PPI team members (including care-home residents); however, although they were offered opportunities to comment on data extraction criteria and papers, the labour-intensive nature of the work, complexity and technical vocabulary meant that it was only possible to involve them meaningfully in discussing the findings. These discussions occurred through meetings and one-to-one conversations about data relating to the two key themes.

## Conclusions

Few studies have attempted to involve older care-home residents in the research process. Nonetheless, some have managed to do this successfully. Older care-home residents have been involved as PPI members in two ways: as collaborators in small-scale participatory action research and as advisors on large studies. Multiple barriers to and facilitators of involving residents as PPI members were identified and can be grouped under social factors, skills, resources, care-home organisational factors and the organisation of the research. All studies involving residents in research have been predominantly qualitative in design. Future research is needed to test whether care-home residents could be successfully involved in research with a quantitative design and/or as collaborators or peer researchers, particularly in large studies involving several care homes.

The reporting of PPI varies. Comprehensive reporting would allow readers to better assess the impact of PPI on the research. While it is difficult at present to evaluate PPI impact in research outcomes, such impact has been demonstrated in more inclusive research processes with care-home residents.

**Key points**

- Older care-home residents have successfully collaborated or advised researchers in a variety of studies.
- Involvement of older care-home residents in the research process has differed depending on the size of the study.
- Involvement of older care-home residents as PPI members has been found in studies with largely qualitative research designs.
- There are multiple facilitators of and barriers to older care-home residents being involved in the research process.

## Supplementary data

Supplementary data mentioned in the text are available to subscribers in *Age and Ageing* online.

## Conflicts of interest

None declared.

## Funding

The research was funded by the National Institute for Health Research (NIHR) Collaboration for Leadership in Applied Health Research and Care East of England (CLAHRC EoE), at Cambridgeshire and Peterborough NHS Foundation Trust. The views expressed are those of the authors and not necessarily those of the NHS, the NIHR or the Department of Health.

## Supplementary Material

Supplementary Data
