# Supplementary material for: Older care-home residents as collaborators or advisors in research: a systematic review
Source: Age Ageing. 2016 Jan 19;45(3):337–45. doi: 10.1093/ageing/afv201 (PMC4846791; doi:10.1093/ageing/afv201)
Supplement: Supplementary Data [file supp_45_3_337__index.html]

Older care-home residents as collaborators or advisors in research: a systematic review — Older care-home residents as collaborators or advisors in research: a systematic review — Older care-home residents as collaborators or advisors in research: a systematic review — Supplementary Data 

# Older care-home residents as collaborators or advisors in research: a systematic review

## Supplementary Data

Supplementary Data

- Supplementary Data - Docx file
