# Supplementary material for: Older care-home residents as collaborators or advisors in research: a systematic review
Source: Age Ageing. 2016 Jan 19;45(3):337–45. doi: 10.1093/ageing/afv201 (PMC4846791; doi:10.1093/ageing/afv201)
Supplement: Supplementary Data [file supp_afv201_afv201supp.docx]

**SUPPLEMENTARY DATA**

**APPENDIX A**

| **ID** |  |
| --- | --- |
| **1, Authors** |  |
| **2, Year of publication** |  |
| **3, Title** |  |
| **4a, Type of publication** |  |
| **4b, Journal name/source** |  |
| **5, Topic of study** |  |
| **6, Aims of study** |  |
| **7, Region** |  |
| **8, Type of care establishment** |  |
| **9, Number of homes involved** |  |
| **10, Funding body** |  |
| **11, Study design** |  |
| **12, Terminology used** |  |
| **13, Type of PPI (e.g. collaboration, consultation)** |  |
| **14, Aims of PPI** |  |
| **15, Groups of people involved** |  |
| **16, Amount of PPI People** |  |
| **17, Demographics of PPI** |  |
| **18, Interaction between PPI groups** |  |
| **19, Recruitment of PPI members** |  |
| **20, Stages of project with PPI involvement** |  |
| **21, Motivation of PPI people to become involved** |  |
| **22, Frequency of involvement** |  |
| **23a, Length of overall involvement** |  |
| **23b, Length of study** |  |
| **24, Location of meetings** |  |
| **25, Methods of involvement** |  |
| **26, Retention of PPI people** |  |
| **27, Evaluation of PPI** |  |
| **28, Perceived impact of PPI** |  |
| **29, Barriers to PPI/ adverse events** |  |
| **30, Elements enhancing PPI** |  |
| **31, Remuneration of PPI people** |  |
| **32, Training provided for PPI people** |  |
| **33, Academic staff skills** |  |
| **34, Other resources needed** |  |
| **35, Transparency of PPI process** |  |
| **36, Match between aims of PPI and outcomes** |  |
| **37, Scale of PPI reporting** |  |
| **38a, Quality of reporting** |  |
| **38b, Quality of study - methodology** |  |
| **39, Other comments** |  |
| **40, decision or advice PPI are involved with** |  |
| **41, Direct voice of residents** |  |

**APPENDIX B**

Systematic review synthesis

| **Themes** | **Data categories involved** |
| --- | --- |
| Study details | 1, Authors  2, Year of publication  3, Title  4a, Type of publication  4b, Journal name/source  7, Region  8, Type of care establishment  10, Funding body |
| Topic and aims of study | 5, Topic of study  6, Aims of study |
| Study design, methodology | 9, Number of homes involved  11, Study design  23b, Length of Study |
| Terminology | 12, Terminology used |
| Type and aims of PPI | 13, Type of PPI  14, Aims of PPI  40, Decision or advice PPI are involved with |
| PPI people and their interaction | 15, Groups of people involved  16, Amount of PPI people  17, Demographics of PPI  18, Interaction between PPI groups  26, Retention of PPI people |
| Recruitment and motivation of PPI members | 19, Recruitment of PPI members  21, Motivation of PPI people to become involved |
| Stages, methods and length of involvement | 20, Stages of project with PPI  22, Frequency of involvement  23a, Length of overall involvement  24, Locations of meetings  25, Methods of involvement |
| Evaluation of PPI  Critical appraisal questions | 27, Evaluation of PPI  28, Perceived impact of PPI  40, Decision or advice PPI are involved with  41, Direct voice of PPI |
| Barriers to PPI | 29, Barriers to PPI |
| Elements enhancing PPI | 30, Elements enhancing PPI |
| Resources needed | 31, Remuneration of PPI people  32, Training provided for PPI people  33, Academic staff skills  34, Other resources needed |
| Quality of reporting  Critical appraisal questions | 35, Transparency of PPI process  36, Match between aims of PPI and outcomes  37, Scale of PPI reporting  38a, Quality of reporting  38b, Quality of study methodology |

**Supplementary Data**

**Full list of references**

1. National Institute of Health Research (2015) *Funding: Patient and Public Involvement.* [Online] Available from: <http://www.nihr.ac.uk/funding/pgfar-patient-and-public-involvement.htm> [accessed 03.06.15].
2. Boyce, M., O’Brien, N., Munn-Giddings, C. and McVicar, A. (2009) How does the rhetoric of ‘user participation’ in research apply to older people? *Research, Policy and Planning*, 27(1), 55-63.
3. Clough, R., Green, B., Hawes, B., Raymond, G. and Bright, L. (2006) *Older people as researchers: Evaluating a participative project*. York: Joseph Rowntree Foundation Report, 1-73.
4. Dewar, B. J. (2005) Beyond tokenistic involvement of older people in research – a framework for future development and understanding. *International Journal of Older People Nursing in association with Journal of Clinical Nursing*, 14(3a), 48–53.
5. Fudge, N., Wolfe, C. D. A. and McKevitt, C. (2007) Involving Older People in Health Research. *Age and Ageing*, 36, 492-500.
6. Gutman, C., Hantmana, S., Ben-Oz, M., Criden, W., Anghel, R. and Ramon, S. (2014) Involving Older Adults as Co-researchers in Social Work Education. *Educational Gerontology*, 40, 186-197.
7. Ross, F., Donovan, S., Brearley, S. et al. (2005) Involving older people in research: methodological issues. *Health and Social Care in the Community*, 13(3), 268–275.
8. Baur, V., Abma, T. and Widdershoven, G. (2010) Participation of marginalised groups in evaluation: Mission impossible? *Evaluation and Program Planning.* 33, 238-245.
9. Davies, S. L., Goodman, C., Manthorpe, J., Smith, A., Carrick, N. and Iliffe, S. (2014) Enabling research in care homes: an evaluation of a national network of research ready care homes. *BMC Medical Research Methodology*, 14(47), 1-8.
10. ENRICH (2013) *Enabling Research in Care Homes,* *A Tool Kit for Care Home Research*. Available at <http://www.enrich.nihr.ac.uk/> [accessed 30.07.15]
11. Froggatt, K. A., Davies, S., Atkinson, L. et al. (2006) The joys and tribulations of partnership working in care homes for older people. *Quality in Ageing - Policy, practice and research.* 7(3), 26-32.
12. Care Quality Commission: The independent regulator of health and social care in England (2015) Website Available at: http://www.cqc.org.uk/content/care-homes [accessed 17.11.15].
13. Gordon, A. L., Franklin, M., Bradshaw, L., Logan, P., Elliott, R. and Gladman, J, R. (2013) Health status of UK care home residents: a cohort study. *Age and Ageing*. 43(1), 97–103.
14. Lievesley, N., Crosby, G., Bowman, C. and Midwinter, E. (2011). *The changing role of care homes.* London: Bupa and Centre for Policy on Ageing.
15. Forder, J. and Fernandez, J-L. (2011) *Length of stay in care homes*, Report commissioned by Bupa Care Services, PSSRU Discussion Paper 2769, Canterbury: PSSRU.
16. Luff, R., Ferriera, Z. and Meyer, J. (2011) *Methods Review 8*: *Care Homes*. London: NIHR School for Social Care Research.
17. Twiddy, M., Muir, D. and Boote, J. on behalf of the PPI in Care Home Research Group (2013) *Public Involvement in Care Home Research,* *Workshop Report*. Yorkshire and the Humber: NIHR Research Design Service.
18. Moher, D., Liberati, A., Tetzlaff, J., Altman, DG. and Group, TP. (2009) Preferred reporting items for systematic reviews and meta-analyses: the PRISMA statement. *PLoS Med*, 6.
19. Hyde, P., Burns, D., Hassard, J. and Killett, A. (2014) Colonizing the Aged Body and the Organization of Later Life. *Organizational Studies.* 35(11), 1699-1717.
20. Hyde, P., Burns, D., Killett, A., Kenkmann, A., Poland, F. and Gray, R. (2014) Organisational aspects of elder mistreatment in long term care. *Quality in Ageing and Older Adults.* 15(4), 197-209.
21. Killett, A., Burns, D., Kelly, F. et al. (2014) Digging deep: how organisational culture affects care home residents’ experiences. *Ageing and Society.* 36(1), 160-188.
22. Hannes, K. (2011) Chapter 4: Critical appraisal of qualitative research. In: Noyes, J., Booth, A., Hannes, K., Harden, A., Harris, J., Lewin, S. and Lockwood, C. (editors), *Supplementary Guidance for Inclusion of Qualitative Research in Cochrane Systematic Reviews of Interventions.* Version 1 (updated August 2011). Cochrane Collaboration Qualitative Methods Group, Available from URL <http://cqrmg.cochrane.org/supplemental-handbook-guidance> [accessed 30.11.15]
23. Chenoweth, L. and Kilstoff, K. (2002) Organizational and structural reform in aged care organizations: empowerment towards a change process. *Journal of Nursing Management.* 10, 235-244.
24. Aveyard, B. and Davies, S. (2006) Moving forward together: evaluation of an action group involving staff and relatives within a nursing home for older people with dementia. *International Journal of Older People Nursing.* 1, 95-104
25. Hewitt, G., Draper, A. and Ismail, S. (2013) Using Participatory Approaches with Older People in a Residential Home in Guyana: Challenges and Tensions. *Journal of Cross Cultural Gerontology.* 28, 1-25.
26. Tadd, W., Woods, R., O’Neill, M. et al. (2011) *Promoting Excellence in All Care Homes (PEACH).* *Final Report.* DoH Policy Research Programme Prevention of Abuse and Neglect in Care of Older Adults (PANICOA).
27. Bowers, H., Clark, A., Crosby, G. et al. (2009) *Older people’s vision for long-term care.* *Final Report.* York: Joseph Rowntree Foundation.
28. Cheek, J., Gilbert, A., Ballantyne, A. and Penhall, R. (2004) Factors Influencing the Implementation of Quality Use of Medicines in Residential Aged Care. *Drugs & Aging.* 21(12), 813-824.
29. Killett, A., Burns, D., Hyde, P., Poland, F., Gray, R. and Kenkmann, A. (2012) *Organizational Dynamics of Respect and Elder Care*. *Final report.* DoH Policy Research Programme Prevention of Abuse and Neglect in Care of Older Adults (PANICOA).
30. Killett, A., Bowes, A., Brooker, D. et al. (2013) *What makes a real difference to resident experience? Digging deep into care home culture: The CHOICE (Care Home Organisations Implementing Cultures of Excellence).* *Final report.* DoH Policy Research Programme Prevention of Abuse and Neglect in Care of Older Adults (PANICOA).
31. Delbecq, A, L. (1983) The nominal group as a technique for understanding the qualitative dimensions of client needs. In: Bell, R. A., Sundel, M., Aponte, J. M. et al., editors. *Assessing health and human service needs*. New York: Human Services Press.
32. Shura, R., Siders, R. A. and Dannefer, D. (2011) Culture Change in Long-term Care: Participatory Action Research and the Role of the Resident. *The Gerontologist.* 51(2), 212-225.
33. Hewitt, G., Draper, A., Ismail, S. and Patterson, S. (2007) Improving food provision in a Guyanese home for the elderly: a participatory approach. *Public Health Nutrition.* 10(6), 552-558.
34. Mitchell, P. and Koch, T. (1997) An attempt to give nursing home residents a voice in the quality improvement process: the challenge of frailty. *Journal of Clinical Nursing.* 6, 453-461.
35. Baur, V. and Abma, T. (2012) ‘The Taste Buddies’: participation and empowerment in a residential home for older people. *Ageing and Society.* 32(6), 1055-1078.
36. Staley, K. (2012) *An evaluation of service user involvement in studies adopted by the Mental Health Research Network*. London: MHRN.
37. Boote, J., Barber, R. & Cooper, C. (2006) Principles and indicators of successful consumer involvement in NHS research: results of a Delphi study and subgroup analysis. *Health Policy.* 75 (3), 280-297.
38. Stocks, S. J., Giles, S, J., Cheraghi-Sohi, S. & Campbell, S, M. (2015) Application of a tool for the evaluation of public and patient involvement in research. *BMJ Open*. (5)3. Available from: <http://bmjopen.bmj.com/content/5/3/e006390.full> [accessed 15.09.15].
39. Davies, S., Powell, A., Aveyard, B. (2002) Developing continuing care: towards a teaching nursing home. *British Journal of Nursing*. 11(20), 1320-1328.
40. Burns, D., Hyde, P., Killett, A., Poland, F. and Gray, R. (2014) Participatory Organizational Research: Examining Voice in the Co-production of Knowledge. *British Journal of Management.* 25(1), 133-144.
41. Killett, A., Hyde, P., Burns, D., Gray, R. and Poland, F. (2013) How organizational factors interact to influence the quality of care of older people in the care home sector*. Journal of Health Services Research & Policy*. 18(Suppl. 1), 14–22.
